# Spatial self-segregation of pioneer cyanobacterial species drives microbiome organization in biocrusts

**DOI:** 10.1038/s43705-022-00199-0

**Published:** 2022-11-16

**Authors:** Corey Nelson, Ana Giraldo-Silva, Finlay Warsop Thomas, Ferran Garcia-Pichel

**Affiliations:** 1 Center for Fundamental and Applied Microbiomics, Biodesign Institute, Arizona State University, Tempe, AZ 85287 USA; 2 School of Life Sciences, Arizona State University, Tempe, AZ 85287 USA

**Keywords:** Soil microbiology, Microbial ecology

## Abstract

Microbial communities are typically characterized by some degree of self-organization. In biological soil crust (biocrust) communities, vertical organization of resident populations at the mm scale is driven by organismal adaptations to physicochemical microniches. However, the extent of horizontal organization and its driving processes are unknown. Using a combination of observational and genetic mapping, we provide evidence for a highly defined, horizontal self-organization (patchiness) at the mm to cm scale in a successionally early biocrust community dominated by the pioneer cyanobacteria, *Microcoleus vaginatus* (Microcoleaceae) and *Parifilum* sp. (Coleofasciculaceae). Experiments with representative isolates of each species demonstrate that the phenomenon is driven by active spatial segregation based on cross-species sensing through the exometabolome acted upon with motility responses. Further, we show that both species share the ability to enrich for specialized cyanospheres of heterotrophic bacteria at smaller scales, and that these cyanospheres are characterized by compositional host-specificity, thus expanding the reach of spatial patchiness beyond primary producers. Our results highlight the importance of specific microbial interactions in the emergence of microbiome compositional architecture and the enhancement of microbial diversity.

## Introduction

Microbial communities assemble into more than stochastic mixtures of its members, displaying marked spatial distribution patterns that constitute one of their emergent, defining traits [1, 2]. This organized species-assembly is perhaps best exemplified in the case of the conspicuously colored layering of microbial mats [3], and in biofilms [4], but is a general phenomenon that has significant functional implications [5]. In many microbiomes, organismal preference for spatially varied microniches or resources (i.e., light extinction with depth or increasingly anoxic underlayers) is thought to contribute strongly to the emergence of these spatial patterns. In addition, microbial interactions can also be at play. Microbial interactions between various heterotrophic bacteria and oxygenic phototrophs (from cyanobacteria to algae and plants) are an example of the latter, wherein specific heterotrophs assemble within the phototroph’s ‘sphere of influence’ [6], in communities often designated as cyanospheres [7–9], algal spheres [10, 11] or the much better studied rhizospheres. While studying such interactions can be challenging due to the species and functional complexity of microbiomes, in some cases the interactions are mediated by specific molecules in the organismal exometabolome [12, 13]. Biological soil crust communities, or biocrusts, represent an exceptional model for the study of microbial community assembly in soil systems as both their diversity and functionality are comparatively well-defined.

Biocrusts are microbial communities commonly found inhabiting the topsoil of dryland ecosystems [14, 15] and are estimated to cover ~12% of the continental surface [16]. As microbial communities of local and global ecological relevance, they contribute significantly to global C and N biogeochemical cycles [17] and provide essential ecosystem services locally, such as erosion control and soil fertilization [16, 18–21]. The successional development of biocrust communities, as well as the factors that determine the vertical distribution of microbial taxa within each successional stage, have been well studied and seem relatively consistent in most dryland locales [22–28]. Filamentous bundle-forming cyanobacteria such as *Microcoleus vaginatus* and members of the family *Coleofasciculaceae* (formerly *Microcoleus steenstrupii* complex) are considered foundational biocrust organisms [29, 30] that facilitate the initial stabilization of bare soils allowing further colonization of other organisms, such as heterocystous cyanobacteria, lichens and bryophytes [31, 32]. Biocrust are vertically organized at the mm scale: heterocystous cyanobacteria inhabit the soil surface [33], non-heterocystous species occupying (and moving within) the top 5 mm [34], ammonia oxidizing bacterial populations peak at some 2.5 mm deep [35], and there is a gradation of functional heterotrophic types that become progressively more oligotrophic in character with depth [24].

In contrast, the horizontal distribution of biocrust communities over small spatial scales (cm- to mm-scale) is underexplored, although high replication sampling shows that they are demonstrably patchy over cm-scale distances [36–38]. The forces influencing the horizontal distribution of phototrophic taxa, whether by similar adaptations to physicochemical microniches or by active processes, remain to be determined. Principally, they could be the result of stochasticity during community assembly, mirror an underlying patchiness in resources, or be the result of more sophisticated microbial interactions. For example, the foundational biocrust cyanobacterium *Microcoleus vaginatus* enters into mutualistic relationships with specific diazotrophic heterotrophs creating a bundle-associated cyanosphere microbiome enriched in nitrogen-fixing and copiotrophic bacteria, while oligotrophic bacteria and other phototrophs are relatively enriched in the bulk soil away from it [7, 39]. This active spatial landscaping by *M. vaginatus* creates heterogeneity at the sub-mm scale. While not yet tested directly, there is evidence that other morphologically similar but genetically distant bundle-forming pioneer cyanobacteria can benefit from similar associations with cyanosphere microbes [40].

Given the central role of bundle-forming filamentous cyanobacteria as both pioneers and microbiome landscapers, we sought to understand the emergence of horizontal patchiness in simple, successionally early cyanobacterial biocrusts dominated by these organisms. We present here the results of a species-explicit mapping of cyanobacterial patchiness at the cm-scale, its influence on bacterial patchiness at the sub mm-scale through the formation of cyanospheres, and the role played by microbial interactions in the self-organization of biocrusts microbiomes in space.

## Methods

### Small-scale surveying and single cyanobacterial bundle sampling

**Fig. 1:**
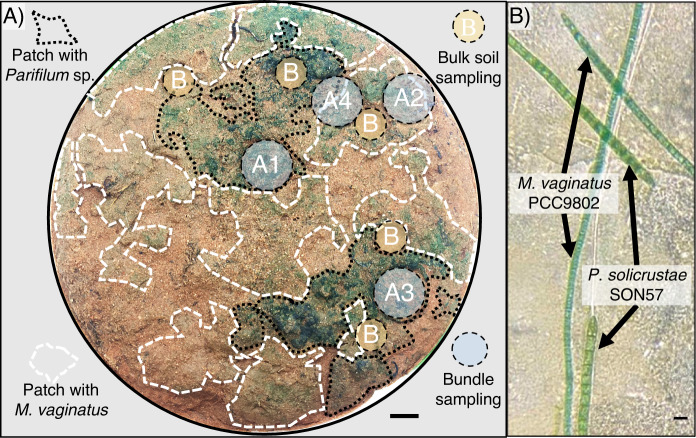
Patchiness in cyanobacterial populations in a cyanobacterial biocrust at the cm scale. **A** Patches attributable to *M. vaginatus* (dashed white lines) and *Parifilum* sp. (dashed black lines) on a rehydrated biocrust are visible through differential coloration. Areas sampled for bulk soil (B) and cyanobacterial bundles (A1-4) are indicated by circles. Scale bar is 1 cm. **B** Light microscopy image showing trichome color and morphology differences between cultured representative strains *M. vaginatus* PCC9802 and *P. solicrustae* SON57 from our culture collection. Scale bar is 10 µm. Images have been optimized in brightness, contrast and color saturation with equal modification for all pixels.

All sampling was conducted within a 15 cm diameter circular sample of cyanobacterial biocrust (Fig. 1A, S1) excised from sandy soils in the Jornada Range (Las Cruces, New Mexico; lat 32.59194°, long −106.85286°) in May 2019, selected because of the co-dominance of populations of two bundle-forming cyanobacteria: *Microcoleus vaginatus* (*Microcolaceae*) and *Parifilum* sp. (*Coleofasciculaceae*), a member of the formerly named “*Microcoleus steenstrupii* complex” [29]. A blind sampling targeting single bundles was carried out across the sample. Single bundles (*n* = 30) were excised from the biocrust by micromanipulation under a dissecting microscope (NIKON SMZ-U, Minato, Tokyo, Japan), following the procedures previously described [7]. Bundles were randomly sampled across the whole plate without prior knowledge or expectation on cyanobacterial identity (blind sampling). This first sampling established the correlation between cyanobacterial identity and patch color. To confirm this observation, spot checks were performed based on differential color patches as well as to delimit an area where both species could be found in close proximity. Each bundle was examined under a compound microscope (Labophot-2, Nikon, Tokyo, Japan) to assess filament morphology and to provide a preliminary taxonomic assignment. These assignments were based on the presence of tapered trichome ends whose taper involved more than one cell and the absence of constrictions at cross-walls (indicating *M. vaginatus*) [41], or the presence of cross-wall constrictions (typically every second cell) and absence of multicellular apical taper (indicating *Parifilum* sp.) [29]. The cyanobacterial bundles were thus categorized as either randomly-sampled *M. vaginatus* (R-*Mv*) and randomly-sampled *Parifilum* (R-*Pf*), and used to construct a map of patches aided by their differential color. Further spot checks by microscopy corroborated the accuracy of this observation.

To study the role of spatial proximity on cyanosphere community composition, we sampled a 1.5 cm diameter area where both cyanobacteria were present. In this sampling, cyanobacterial bundles were categorized as collocated *M. vaginatus* (C-*Mv*) and collocated *Parifilum* sp. (C-*Pf*). Additionally, five soil cores were taken for determination of bulk soil community composition by coring with 1 cm diameter metal corers to a depth of 5 mm.

### Cyanobacterial segregation assays

Culture suspensions of previously isolated strains of each species were used here: *M. vaginatus* PCC9802, in axenic state, originally isolated from biocrusts in the Colorado Plateau, and available through the Pasteur Culture Collection of Cyanobacteria (Paris, France) and *Parifilum solicrustae* SON57, in unicyanobacterial state, isolated from Sonoran desert biocrusts [42], available through The University of Texas Culture Collection (UTEX ZZ1246). Cultures were grown in liquid BG11 medium [43] and homogenized by repeated passage through a syringe [42], adjusted to similar concentration visually, combined in equal volumes, mixed, and deposited onto solid BG11 + 2% agar Petri plates to form 1.5 cm diameter spots (*n* = 30). Plates were incubated at 23 °C, under 18–20 μE m^−2^ s^−1^ of white light and with a 14 h illumination/10 h dark cycle (standard conditions). The cyanobacterial trichomes in the spots were examined through at 400x magnification under a compound microscope (Leica DM500, Wetzlar, Germany) immediately after deposition, and after 4.5 and 9 days of incubation. The number of microscopic fields (*n* = 300 microscope fields; 10 per spot) where both *M. vaginatus* and *P. solicrustae* trichomes were observed, were scored as “mixed”, and fields where only a single species was present or overwhelmingly dominant were scored as “segregated”. The average number of trichomes counted per field was 30. For statistical assessment, non-parametric tests (Kruskal-Wallis with Dunn post-hoc) were performed given the lack of certainty in the normality of the data (normal according to D’agostino Pearson test, but not normal according to Shapiro test).

To determine if self-segregation of cyanobacterial strains was mediated by exuded exometabolites, the chemotactic response of *M. vaginatus* to the spent medium of *P. solicrustae* was tested. Spent medium for each cyanobacterial strain was prepared by culturing in BG11 medium for 14 days under standard conditions, clearing of biomass by centrifugation (5000 rpm for 10 min) and sterilizing the supernatant through a 0.2 μm syringe filter. Spent media were kept frozen until use. Sterile cellulose disks were dipped in either spent medium of *P. solicrustae* SON57, spent medium of *M. vaginatus* PCC9802 or BG11 medium as controls, and placed in the center of solid BG11 + 2% agar plates (*n* = 5). *M. vaginatus* PCC9802 was then inoculated at 0.5 or 1 cm from the disks and incubated under standard conditions for 9 days. Tendrils of filaments produced by *M. vaginatus* were photographed, the area covered as a function of orientation measured using ImageJ [44], and tested for spatial distribution (towards or away from cue) using a *t* test. Data for the 0.5 cm distance was distorted by the physical proximity of the cellulose disk and was not used. No equivalent experiment quantifying the reaction of *P. solicrustae* SON57 to *M. vaginatus* PCC9802 growth medium was performed as *P. solicrustae* does not produce macroscopic tendrils in culture. For statistical assessment, *t* tests were performed after ensuring that data were normally distributed.

### DNA extraction and 16S rRNA gene sequencing

Single bundle and bulk soil community DNA was extracted with the Powersoil Pro extraction kit (QIAGEN, Hilden, Germany) following manufacturer’s instructions for downstream processing. Single cyanobacterial bundles typically have low DNA yields, which makes downstream analyses prone to environmental contamination signals. To account for this, seven sterilized cotton sewing threads similar in size to bundles were subject to sampling, cleaning, extraction, and sequencing procedures identical to those carried out for cyanobacterial bundles, serving as controls for operator and environmental contamination as previously described [7]. Microbial community structure was determined through next-generation sequencing of 16S rRNA genes. The V4 region of 16S rRNA genes was amplified using general bacterial primers 515 F/806 R. PCR was performed in triplicate, products were pooled, and PCR protocols were performed as previously described [45]. 240 ng of PCR product per sample were pooled and cleaned using the QIA Quick PCR Purification kit. DNA library concentration was quantified by qPCR using the ABI Prism^®^ kit, brought to final concentration of 4 nM, denatured, and diluted again to a final concentration of 4 pM. 180 μL of PhiX (Illumina) at a concentration of 12 pM and 150 μL of buffer HT1 (Illumina) were mixed with 270 μL of the pooled library and loaded in the MiSeq Illumina sequencer, adding custom 16S rRNA sequencing primers [45] on a paired-ends sequencing flow cell 2 (2 × 250 bp).

### Bioinformatic analyses

Paired-end reads obtained from Illumina sequencing were demultiplexed, and quality controlled using the DADA2 plugin [46] available in Qiime2 version 2020.11 [47], creating a feature table containing representative sequences (features) and their frequency of occurrence. The resulting feature table was filtered out to remove rare features. Only features that were present in at least 3 samples were kept. Highly variable positions were removed using MAFFT [48], and phylogenetic trees were generated using FastTree [49]. Preliminary taxonomic assignments were done with the RDP (Ribosomal Database Project) classifier [50], and representative sequences were then aligned against the Greengenes 13_8 database core reference alignment [51]. Cyanobacterial and non-cyanobacterial sequences were then split into separate feature tables [52] and the cyanobacterial features were further curated via Cydrasil3 [53] (https://www.cydrasil.org). The feature table containing non-cyanobacterial sequences represented the “cyanosphere” of bundles or the non-cyanobacterial community of bulk soil. Sequences that were present in at least three of the seven cotton thread controls and exhibited counts higher than those in bundle communities were considered contaminants and removed from feature tables. In order to compare species composition among our microbial communities of interest (groups: community of bulk soil, randomly-sampled *M. vaginatus* (R-*Mv*), randomly-sampled *Parifilum* (R-*Pf*), collocated *M. vaginatus* (C-*Mv*) and collocated *Parifilum* sp. (C-*Pf*)) principal coordinate analysis (PCoA) was performed in R, based on Bray-Curtis pairwise distances calculated from the Hellinger transformed data [54]. Significance between groups was tested on the Bray-Curtis matrix using the ADONIS test included in the R vegan package [55]. Purity of cyanobacterial bundles was determined by calculating the proportion of cyanobacterial reads associated to the cyanobacterial host for each bundle. Differences in purity of *M. vaginatus* and *Parifilum* sp. bundles were tested for significance with a non-parametric Wilcoxon rank sum test using R [54].

### Non-cyanobacterial community composition

Taxonomy was assigned to the top 85% most abundant ASV reads in cyanosphere and non-cyanobacterial community of bulk soil using NCBI blastn [56], to genus-level when possible. Literature searches were conducted to categorize bacterial taxa as either oligotrophic, copiotrophic, unclear, or unknown. Genera were binned into these categories if two or more sources described them as such based on environmental distribution or physiological experiments. The differences in total proportion of oligotrophic vs. copiotrophic reads were pooled and tested for significance with a non-parametric Kruskal-Wallis test, with Dunn post-hoc tests.

### Nitrogen fixation potential

Nitrogen fixation potential of cyanospheres and bulk soil communities was evaluated using quantitative real-time PCR (qPCR) to quantify the gene copy number of 16S rRNA (a proxy for bacterial biomass) and *nif*H (a proxy for nitrogen fixation capacity) genes of the appropriate community DNA extracts, as previously described [7, 39]. For 16S rRNA gene quantitation, a universal (bacterial/archaeal) primer set (338 F 5'- ACTCCTACGG GAGGCAGCAG -3', 518 R 5'- GTATTACCG CGGCTGCTGG -3') [57] was used. For *nif*H quantitation, a standard primer set for *nif*H (PolF 5'- TGCGAYCCSA ARGCBGACTC-3', PolR 5’-ATSGCCATCA TYTCRCCGGA-3') [58] was used. The PCR reactions were performed in triplicate using PerfeCTa SYBR Green FastMix (Quantabio, Beverly, MA, USA) in an ABI ViiA 7 thermocycler (Applied Biosystems, Foster City, CA, USA) under conditions previously published [59]. Nitrogen fixation potential for each bundle or bulk soil community assessed as the ratio of *nif*H to16S gene copy numbers detected. Ratios were log transformed to comply with normality and variance homogeneity before testing for significance with a one-way ANOVA using R [54]. 16S rRNA and *nif*H gene quantitation were also performed on DNA extracts from cotton thread controls to determine if contaminants had an impact on nitrogen fixation potentials.

## Results

### Small-scale spatial patterns

We observed a pattern of patches with differential coloration of the cyanobacterial biomass upon inspection of a rehydrated 15 cm diameter sample under the dissecting scope: some patches appearing more olive green and others more blue-green (Fig. 1A, dashed white and black lines, respectively). To test if this observation reflected differential species dominance, we sampled and analyzed single cyanobacterial bundles by micromanipulation, both morphologically (through examination under the compound microscope) and genetically. Out of 45 single cyanobacterial bundles excised and analyzed from an area of some 176 cm^2^, there was 100% congruence in taxonomic assignments between morphological and genetic approaches. We also found a nearly absolute coincidence between areas with olive-green appearance and the dominance of *M. vaginatus* bundles, as well as blue-green patches containing exclusively *Parifilum* sp.-dominated bundles (Fig. 1A, spots A1-4; Table S1). This demonstrated the presence of a structured, horizontal patchiness pattern in species composition of the main primary producers. Because blue-green and olive-green patches had defined borders and did not overlap, we conclude that the two species were spatially neatly segregated at this small scale.

**Fig. 2:**
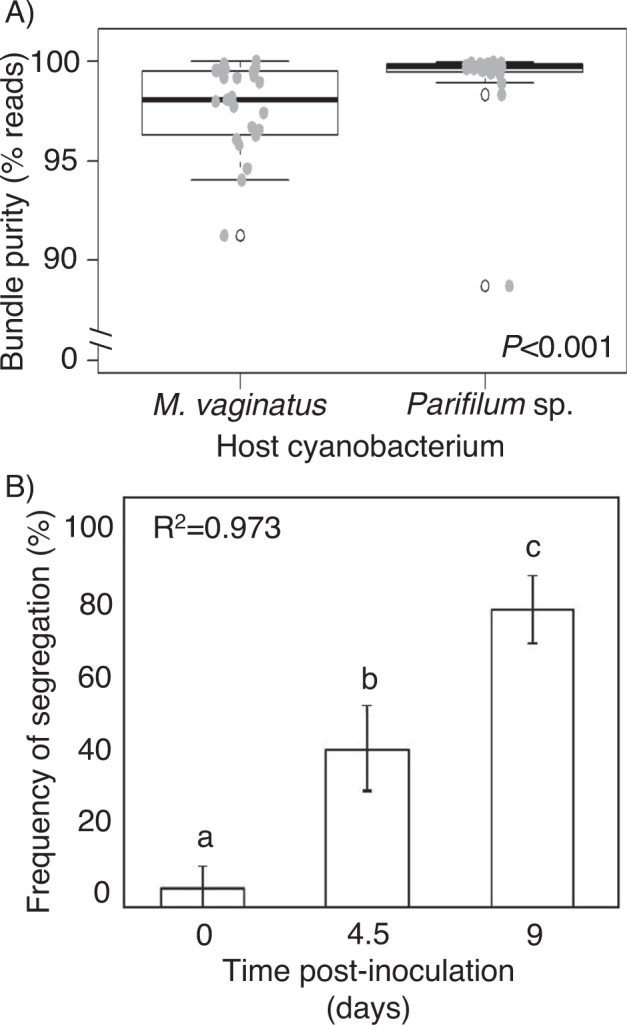
Self-segregation of pioneer cyanobacteria in natural bundle populations and culture segregation assays. **A** Boxplot of the proportion of cyanobacterial reads contributed by the dominant cyanobacterium in each bundle community. **B** Observed frequencies of segregation in mixed populations of *M. vaginatus* PCC9802 and *P. solicrustae* SON57 over 9 days (*n* = 300 observations per timepoint). Frequencies of segregation increased linearly and were significant at each timepoint according to non-parametric Kruskal-Wallis with Dunn post-hoc tests (*P* < 0.0005 for both).

Below the mm-scale, within single bundles, segregation of the cyanobacterial community composition based on 16S rRNA gene sequencing was also patent: on average, more than 99% of cyanobacterial reads in *Parifilum*- bundles were assignable to *Parifilum* sp., and more than 95% of reads obtained from *Microcoleus*-dominated bundles were assignable to *M. vaginatus* (Fig. 2A) (PERMANOVA, *P* < 0.001). A Wilcoxon rank sum test for ratios showed that *Parifilum* sp. bundles (*n* = 21) contained significantly lower proportions of other cyanobacteria than *M. vaginatus* bundles (*n* = 24) (*P* < 0.001). Further, in *Parifilum*-dominated bundles, less than 0.4% of average reads were assignable to *M. vaginatus*, and in *M. vaginatus*-dominated bundles, only 0.9% matched *Parifilum* sp.

### Active segregation of cyanobacterial isolates and motility responses

To test if segregation was a result of active organismal interactions (as opposed to a legacy of stochastic processes of community assembly and/or competitive exclusion), we conducted assays using isolated strains that had been selected as representative of each species with the expectation that these phototrophs would also then actively self-segregate when co-cultivated under laboratory conditions. These strains were considered representative of field populations based on matched 16S rRNA sequences to existing field populations (they were genetically pedigreed), and that they had been isolated originally from similar early-stage biocrusts, albeit not from the same sample. Immediately after experimental mixing of the two strains and deposition on solid media, microscopic examination revealed that only 5% of fields contained either *M. vaginatus* or *P. solicrustae* trichomes, while 95 % contained mixtures. The frequency of segregation significantly increased at each time point: increasing to 42% after 4.5 days of incubation and reaching 79 % after 9 days (Fig. 2B) (Kruskal-Wallis with Dunn post hoc test, *P* < 0.005 for both). While segregation could have been attained through either motility responses leading to active separation or by allelopathic effects leading to suppression of growth of non-selves, that segregated patches of either cyanobacterium were detected with similar frequency supports the former mechanism. Additionally, the former mechanism would have resulted in linear, progressive separation, whereas the latter would have resulted in exponential segregation dynamics. A linear fit best explains the data in Fig. 2B (after angular transformation for percentages, R^2^ = 0.95) speaking also for segregation being a function of microbial interactions involving motility responses.

**Fig. 3:**
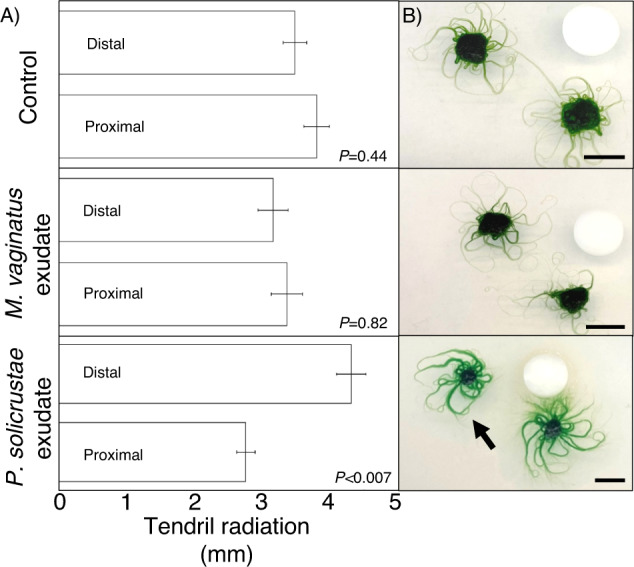
Chemotactic response of *M. vaginatus* PCC9802 to cyanobacterial exudates. **A** Average tendril radiation toward (proximal) or away (distal) stimulus source (*n* = 5). **B** Representative photographs of the chemotactic behavior of *M. vaginatus*. Control consists of BG11 medium containing no exudates. Black arrow indicates *M. vaginatus* tendrils sweeping away from cellulose disk soaked in *P. solicrustae* spent growth medium. Scale bar is 5 mm. Given that the original data were normally distributed, significance of the comparisons is based on *t* test.

We then tested the chemotactic response of *M. vaginatus* to the spent growth medium of *P. solicrustae* using cellulose disk assays. *M. vaginatus* produces bundle-like outgrowths that can move and steer on the surface of the plate (tendrils). We measured the directionality of these tendrils towards (proximal) or away (distal) from the test disk and found that placement of *M. vaginatus* inoculum 1 cm away from the disk offered clearest results. Tendril development of *M. vaginatus* was significantly less developed on the proximal compared the distal side of *P. solicrustae* spent medium (Fig. 3A) (t-test, *P* < 0.007), indicating a strong negative chemotactic effect on the tendrils of *M. vaginatus* when in proximity to the cue source. Many of the tendrils that started from the proximal side in fact appeared to eventually steer away from the disk. None of these responses were seen when using minimal medium (BG11) or *M. vaginatus’* own exudates as controls solutions (t-test, *P* = 0.44 & 0.82, respectively) (Fig. 3B). Thus, we could confirm that motility responses are behind the active segregation of *M. vaginatus* from *P. solicrustae*. The fact that the ability of one strain to react to the other’s exudates remained even when the spent medium was not collected *in natura*, but under culture conditions, speaks for the sensory capacities to target metabolites that are constitutively produced.

### Cyanospheres and bulk soil communities

**Fig. 4:**
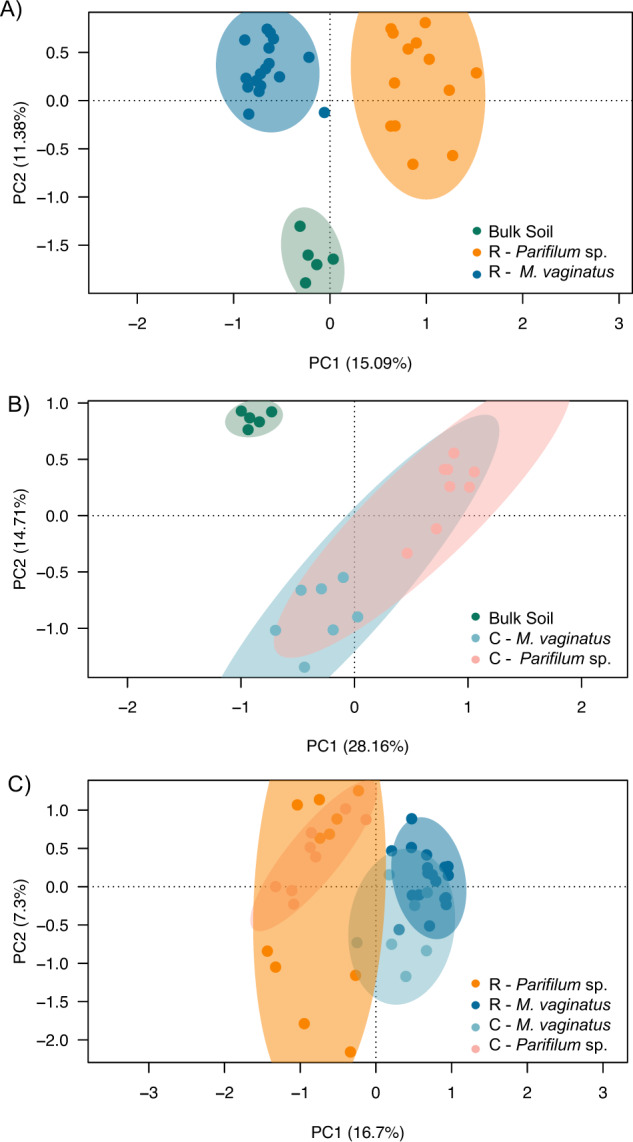
Divergence in community composition of non-cyanobacterial cyanospheres and bulk soil. Principal component analysis based on Bray-Curtis pairwise distances calculated from Hellinger transformed tables containing sequences and their frequency of occurrence, with 95% confidence ellipses drawn for each community. **A** Comparison of bulk soil (*n* = 5), randomly-sampled *Parifilum* sp. (R-*Pf*; n = 13) and randomly-sampled *M. vaginatus* bundles (R-*Mv*; *n* = 17). **B** Comparison of bulk soil (*n* = 5) and *M. vaginatus* (C-*Mv*; *n* = 7) and *Parifilum* sp. (C-*Pf*; *n* = 8) sampled the same cm-scale patch. **C** Comparison of cyanospheres from all sampling groups (randomly-sampled *Parafilum* sp., randomly-sampled *M. vaginatu*s, collocated *M. vaginatus* and collocated *Parifilum* sp.).

To investigate whether observed cyanobacterial patchiness extends to affect cyanosphere composition, we first had to determine if *Parifilum* sp., like *M. vaginatus* [7], maintains a bundle-associated cyanosphere community distinct from that of bulk soils, and also possibly distinct from those of *M. vaginatus’* cyanosphere. For this, we compared the non-cyanobacterial community composition of *Parifilum* sp. bundles randomly excised across the plate in its own patches (R-*Pf*; *n* = 13), to that of bulk soil (*n* = 5) and randomly-sampled *M. vaginatus* (R-*Mv*; *n* = 17). A PCoA ordination of the beta-diversity Bray-Curtis metric on the Hellinger transformed data, provides evidence that *Parifilum* sp. does maintain a “cyanosphere” compositionally differentiated from both the community in the bulk soil (Adonis, F = 4.24, *P* < 0.001) and the cyanosphere of *M. vaginatus* (Adonis, F = 5.04, *P* < 0.001) (Fig. 4A). The differentiation between cyanosphere communities could be ascribed to either a specificity of recruitment patterns on the part of each cyanobacterium (or a preference for either cyanobacterium by certain heterotrophs), or, alternatively, it may be the consequence of the two species occupying spatially segregated patches, thus recruiting from a pool of already differentiated biodiversity existing in those patches. To test if the host cyanobacterium determines the cyanosphere composition, we compared the cyanospheres of bundles of each cyanobacterial species from a subset of bundles collocated in a single, common patch collocated bundles of *M. vaginatus* (C-*Mv*; *n* = 7; spot A4 in Fig. 1A) and collocated *Parifilum* sp. bundles (C-*Pf*; *n* = 8; spot A4 in Fig. 1A), and found that the two cyanospheres were still well differentiated (Fig. 4B; Adonis, F = 5.81, *P* < 0.001). To test if the local community of heterotrophs available for recruitment in the bulk soil also influences cyanosphere composition, we compared the two cyanospheres obtained in randomly-sampled (R) vs. collocated bundles (C). This influence was statistically supported as well: (Fig. 4C; Adonis, F = 4.23, *P* < 0.001). Thus, the composition of cyanospheres in both cyanobacterial species was dependent on both host-specific factors as well as composition of surrounding bulk soil. A PERMANOVA analysis corroborated the influence of both factors and the presence of strong interactions between them: host cyanobacterium (*M. vaginatus* or *Parifilum* sp.; Pseudo-F = 7.4, *P* = 0.0001; Table S3), local community of heterotrophs (randomly-sampled (R) or collocated (C); Pseudo-F = 2.6, *P* = 0.0001; Table S3) and their interaction (Pseudo-F = 1.9, *P* = 0.001; Table S3).

### Functional analyses of non-cyanobacterial communities

**Fig. 5:**
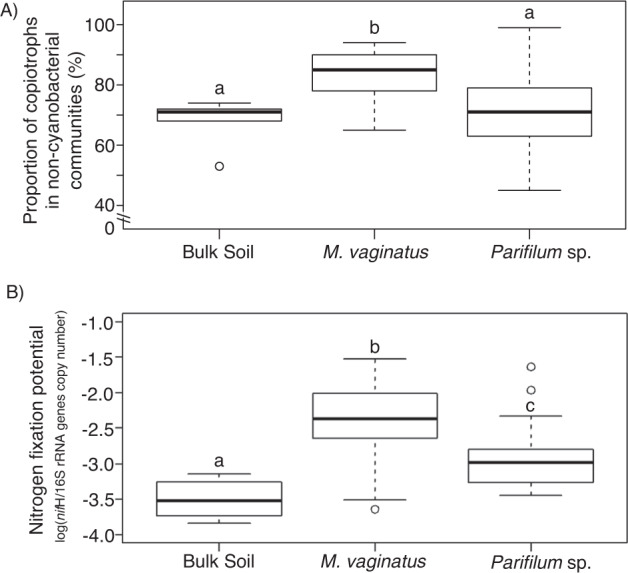
Trait-based analysis of cyanosphere communities. **A** Proportion of copiotrophic taxa in bulk soil, *M. vaginatus* and *Parifilum* sp. cyanospheres (*n* = 5, 24 and 21, respectively). Communities associated with *M. vaginatus* contained higher proportions of copiotrophs than either bulk soil or *Parifilum* sp. communities (*P* < 0.006 for both), while bulk soil and *Parifilum* sp. communities had similar proportions (*P* = 0.449), according to non-parametric Kruskal-Wallis tests, with Dunn post-hoc tests. **B** N_2_-fixation potential of the same non-cyanobacterial microbial communities as in (**A**), determined by their ratio of *nif*H to 16 S rRNA genes copy number. While *M. vaginatus* and *Parifilum* sp. communities had higher nitrogen fixation potentials than bulk soil (*P* < 0.001 and 0.06, respectively, according to ANOVA with Tukey post hoc tests, on log-transformed data to ensure normality), *M. vaginatus* communities had a significantly higher potential than *Parifilum* sp. (*P* < 0.02).

Functionally, the cyanospheres of *M. vaginatus* analyzed in the past [7, 39] were consistently characterized by an enrichment of diazotrophic, copiotrophic heterotrophs over those found in bulk soil. To test if this also held for *Parifilum* sp. cyanospheres, we carried out trait-based inspection of the taxonomically resolved community compositions. *M. vaginatus* cyanospheres contained a significantly larger proportion of copiotrophs (83 ± 8%; *n* = 24) than bulk soil (68 ± 8%; *n* = 5) or the proportion found in *Parifilum* cyanospheres (71 ± 14%; *n* = 21) (Kruskal-Wallis with Dunn post hoc tests, *P* ≤ 0.006 for both). The proportion of copiotrophs found in *Parifilum* sp. cyanospheres, while slightly larger, did not differ from that of bulk soil (Fig. 5A) (Kruskal Wallis with Dunn post hoc, *P* = 0.449). With respect to N-fixation potential, *nif*H/16 S gene copy number ratios were significant higher in both *M. vaginatus* and *Parifilum* cyanospheres over bulk soil (ANOVA plus Tukey post hoc tests, *P* < 0.001 and 0.06, respectively) (Fig. 5B). However, *M. vaginatus* cyanospheres were five-fold more intensely enriched than those of *Parifilum* sp., and the difference between these two cyanospheres was also significant (Tukey, *P* < 0.02). Quantitation of 16S rRNA and *nif*H genes for cotton thread controls were below detection limits, indicating that background contaminants did not contribute to our determination of N-fixation potentials.

## Discussion

### Biocrusts are patchwork microbiomes

We documented how cyanobacterial biocrust are characterized by a sharply delimited, patchy primary producer distribution at the cm scale that involves near complete segregations. This phenomenon has been described for lichen biocrusts, where lichen thalli of different species, approaching cm in size, do not typically overlap [60]. Thus, lateral patchiness as an organizing trait is applicable to biocrusts regardless of compositional or successional stage, including early successional types. The descriptive findings of this study stem from a single sample, but we have seen similar patchiness in other samples of the same locale, and in other locales on the basis of color appearance and microscopic examinations, indicating that this is not a unique phenomenon associated with this particular sample. This observed cyanobacterial biocrust patchiness pattern could not simply be the result of stochastic, foundational patterns, because the lateral expansion rates of biocrust bundle-forming cyanobacteria can reach 1–2 cm per rainy season [61], likely to quickly blur traces of original patterns in settlement. Therefore, segregation through active exclusion or competition for resources must be at play. Our culture experiments confirmed that the former mechanism, is at least partly, behind this phenomenon (Fig. 3). It could rightfully be argued that the cultured strains used for confirmation experiments might not have been representative of the field populations despite having been genetically matched and originally isolated from similar soil crusts. However, it would be highly non-parsimonious to expect that they would self-segregate simply by chance.

Due to the global extent of biocrusts, it is possible to document lateral patterns of species distribution at multiple scales for several types of organisms and to make informed assessments of the forces that shape them. At the global or continental scales, niche separation for climatic parameters imposes spatial segregation of taxa dominance in cyanobacteria, chemolithotrophs and fungi [62–66], whereas at landscape scales community divergence of cyanobacteria and heterotrophs seems to be a function of edaphic variability [67]. Here, we showed that at the cm and smaller scales microbial interactions are involved in this lateral shaping and can be both positive (as in mutualistic cyanosphere formation) and negative (as in segregation of functional competitors within bundles and in patches) in nature.

In addition, the emergent patchiness is not without practical consequences for experimental design in biocrust research in that the common approach of using cm-sized samples in molecular and biochemical analyses will predictably suffer from low precision and statistical power unless high numbers of replicates are obtained, or replicates are combined before analyses. In hindsight, these sampling effects had been demonstrated experimentally early on [36, 37]. On the other hand, that the patchiness in primary producers is associated with differences in color, provides opportunities for remotely sensing these patterns [68] to probe its generalized occurrence or to determine the influence of biocrust heterogeneity up to the meter scale and beyond.

### Interactions between pioneer cyanobacteria as drivers of biocrust architecture

This observed “habitat fragmentation” could, in principle, either be the result of founder effects, specific adaptations to patchiness in resources, or microbe-to-microbe interactions, including interactions between the two cyanobacterial players or differential interactions with further biotic agents (like predators or phages). Field observations and physiological experiments carried out with representative isolates allowed us to link the emergence of spatial patterns to the ability of the cyanobacteria to actively segregate through sensory mechanisms actuating on motility responses. The direct evidence for a negative chemotactic response of *M. vaginatus* to the exudates of *P. solicrustae* provides an explanation for the segregation patterns observed in nature and in culture. Chemotaxis to chemical cues from other organisms, described previously in other cyanobacteria [69], implies an ability to detect specific effector molecules and speaks for sophisticated genetic mechanisms that are much harder to predict than simpler chemotactic responses to metabolic byproducts like oxygen or carbon dioxide. It also adds to a repertoire of photo- and hydrotactic capacities that help *M. vaginatus* cope with changing conditions in biocrusts [34, 70]. However, we could not yet demonstrate a reciprocal response in *P. solicrustae* because of rather mundane experimental constraints like the inability of this strain to form tendrils and its poor performance on plates when exposed to organics. Motility responses are increasingly being recognized as mechanisms for microbiome spatial organization in a variety of systems [71, 72].

### *Parifilum* sp. as sub-par microbiome landscapers

We demonstrate that *Parifilum* sp. maintains a cyanosphere of heterotrophic bacteria compositionally differentiated from that of bulk soil and, in this regard, behaves much like *M. vaginatus* [7, 39], one of its competitors as biocrust pioneers. This parallelism seems to extend to the functional potential of the cyanospheres as it pertains to its diazotrophic potential, although not significantly so with respect to the presence of copiotrophs. In *M. vaginatus*, this cyanosphere leads demonstrably to “C-for-N” mutualistic exchanges that ensure the proliferation of both partners under N-limitation [39]. Hence, we see it as likely that this is also true for *Parafilum* sp. Given that the two cyanobacteria are only distantly related, the capacity to lure a community of diazotrophs would represent a case of convergent evolution that adds to other fascinating convergent traits among bundle-formers, like the capacity to build multi-filament bundles [18] itself, or the ability to migrate vertically in the soil in response to pulses of water availability [34]. Convergent traits in species that do not share a common recent ancestor, but share a defined habitat, speak for their fitness value in the biocrust environment. Perhaps because of convergent evolution, the cyanospheres of *M. vaginatus* and *Parifilum* sp. are not completely equivalent, either compositionally or functionally. Even when recruiting from the same bulk soil populations, a degree of host-specificity in cyanosphere composition can be clearly detected. From the enrichment factors attained with respect to copiotrophs and diazotrophs, it seems that *Parifilum*’s mechanisms of cyanosphere recruitment and maintenance are subpar to those of *M. vaginatus*. Comparative analysis of beta-diversity patterns (Table S4) showed that *M. vaginatus* exerts tighter control over spatial variance in cyanosphere composition than does *Parifilum* sp. However, in spite of this cyanosphere community divergence, there is some experimental evidence from natural communities that beneficial heterotrophs isolated from *M. vaginatus* cyanospheres can interact positively with other bundle-forming species in the Coleofasciculaceae (*Allocoleopsis* sp.), promoting their growth [40]. Apparently, there is some degree of mechanistic cross-host specificity among the cyanosphere mutualists.

### Potential functional impacts of a patchwork biocrust

While we have not directly explored the consequences of patchiness for biocrust function, patchiness is theoretically an important characteristic of various ecological outcomes [73] and could be central to other interactions like predator-prey dynamics [74]. For example, *P. solicrustae* strains show demonstratable resistance to predation by the biocrust predatory prokaryote, *Cyanoraptor togatus* [75], unlike highly-vulnerable *M. vaginatus*. A heterogeneous distribution of cyanobacterial pioneers may be key to slowing the lateral spread of *C. togatus* epidemics, patches of *Parifilum* indirectly protecting neighboring patches of *M. vaginatus*. Because the temperature and precipitation niches of the two cyanobacteria is also differential [65], biocrust function may also show patchiness, with areas dominated by *M. vaginatus* being more productive under colder conditions, and more resilient to long droughts [76], thus ensuring that a biocrust of higher diversity maintains higher functional stability under a varying environment, as ecological theory would predict [77]. Finally, if differences in color (and albedo) were to be sufficient, organismal patchiness may translate into patches in equilibrium surface temperatures under insolation [59].

## Conclusion

By surveying the cm- and sub-mm heterogeneity in the distribution of pioneer cyanobacteria and their associated heterotrophs, we demonstrated that foundational bundle-forming cyanobacteria tend to horizontally segregate and form patches of single cyanobacterial dominance within biocrust communities. We provided evidence that such patchiness is achieved through active cyanobacterial segregation based on motility responses, and that because both cyanobacteria maintain distinct cyanosphere microbiomes, this segregation extends to heterotrophic communities. Self-segregation of foundational cyanobacteria thus acts as a mechanism of spatial self-organization within biocrust microbiomes with potential functional consequences.

## Supplementary Information

Supplementary Information

Table S2

## Data Availability

Raw sequence data have been submitted to NCBI and are publicly available under the BioProject number PRJNA901049.

## References

[1] Valm AM, Welch JLM, Rieken CW, Hasegawa Y, Sogin ML, Oldenbourg R (2011). Systems-level analysis of microbial community organization through combinatorial labeling and spectral imaging. Proc Natl Acad Sci.

[2] Acinas SG, Klepac-Ceraj V, Hunt DE, Pharino C, Ceraj I, Distel DL (2004). Fine-scale phylogenetic architecture of a complex bacterial community. Nature.

[3] Nubel U, Garcia-Pichel F, Clavero E, Muyzer G (2000). Matching molecular diversity and ecophysiology of benthic cyanobacteria and diatoms in communities along a salinity gradient. Environ Microbiol.

[4] Ferrer MD, Mira A (2016). Oral biofilm architecture at the microbial scale. Trends Microbiol.

[5] Vidakovic L, Singh PK, Hartmann R, Nadell CD, Drescher K (2018). Dynamic biofilm architecture confers individual and collective mechanisms of viral protection. Nat Microbiol.

[6] Graham LE, Graham JM, Wilcox LW, Cook ME, Arancibia-Avila P, Knack JJ (2018). Evolutionary roots of plant microbiomes and biogeochemical impacts of nonvascular autotroph-microbiome systems over deep time. Int J Plant Sci.

[7] Couradeau E, Giraldo-Silva A, De Martini F, Garcia-Pichel F (2019). Spatial segregation of the biological soil crust microbiome around its foundational cyanobacterium, *Microcoleus vaginatus*, and the formation of a nitrogen-fixing cyanosphere. Microbiome.

[8] Pascault N, Rué O, Loux V, Pédron J, Martin V, Tambosco J (2021). Insights into the cyanosphere: capturing the respective metabolisms of cyanobacteria and chemotrophic bacteria in natural conditions?. Environ Microbiol Rep.

[9] Vilo C, Dong Q, Galetovic A, Gómez-Silva B (2022). Metagenome-assembled genome of *Cyanocohniella* sp. LLY from the cyanosphere of llayta, an edible andean cyanobacterial macrocolony. Microorganisms.

[10] Califano G, Kwantes M, Abreu MH, Costa R, Wichard T (2020). Cultivating the macroalgal holobiont: effects of integrated multi-trophic aquaculture on the microbiome of *Ulva rigida* (Chlorophyta). Front Mar Sci.

[11] Burke C, Thomas T, Lewis M, Steinberg P, Kjelleberg S (2011). Composition, uniqueness and variability of the epiphytic bacterial community of the green alga *Ulva australis*. ISME J.

[12] Kessler RW, Weiss A, Kuegler S, Hermes C, Wichard T (2018). Macroalgal-bacterial interactions: Role of dimethylsulfoniopropionate in microbial gardening by *Ulva* (Chlorophyta). Mol Ecol.

[13] Segev E, Wyche TP, Kim KH, Petersen J, Ellebrandt C, Vlamakis H (2016). Dynamic metabolic exchange governs a marine algal-bacterial interaction. Elife.

[14] Belnap J, Weber B, Büdel B Biological Soil Crusts as an Organizing Principle in Drylands. In: Weber B, Büdel B, Belnap J (eds). *Biological Soil Crusts: An Organizing Principle in Drylands*. 2016. Springer International Publishing, Cham, pp 3–13.

[15] Belnap J, Büdel B, Lange OL Biological Soil Crusts: Characteristics and Distribution. *Biological Soil Crust: Structure, Function, and Management*. 2001. Springer-Verlag, Berlin, pp 3–30.

[16] Rodriguez-Caballero E, Belnap J, Büdel B, Crutzen PJ, Andreae MO, Pöschl U (2018). Dryland photoautotrophic soil surface communities endangered by global change. Nat Geosci.

[17] Housman DC, Powers HH, Collins AD, Belnap J (2006). Carbon and nitrogen fixation differ between successional stages of biological soil crusts in the Colorado Plateau and Chihuahuan Desert. J Arid Environ.

[18] Garcia-Pichel F, Wojciechowski MF (2009). The evolution of a capacity to build supra-cellular ropes enabled filamentous cyanobacteria to colonize highly erodible substrates. PLoS One.

[19] Belnap J, Gillette D (1997). Disturbance of biological soil crusts: Impacts on potential wind erodibility of sandy desert soils in southeastern Utah. L Degrad Dev.

[20] Zhang YM, Wang HL, Wang XQ, Yang WK, Zhang DY (2006). The microstructure of microbiotic crust and its influence on wind erosion for a sandy soil surface in the Gurbantunggut Desert of Northwestern China. Geoderma.

[21] Gaskin S, Gardner R (2001). The role of cryptogams in runoff and erosion control on Bariland in the Nepal middle hills of the Southern Himalaya. Earth Surf Process Landforms.

[22] Garcia-Pichel F, Belnap J (1996). Microenvironments and microscale productivity of cyanobacterial desert crusts. J Phycol.

[23] Hu C, Zhang D, Huang Z, Liu Y (2003). The vertical microdistribution of cyanobacteria and green algae within desert crusts and the development of the algal crusts. Plant Soil.

[24] Garcia-Pichel F, Johnson SL, Youngkin D, Belnap J (2003). Small-scale vertical distribution of bacterial biomass and diversity in biological soil crusts from arid lands in the colorado plateau. Microb Ecol.

[25] Pombubpa N, Pietrasiak N, De Ley P, Stajich JE (2020). Insights into dryland biocrust microbiome: geography, soil depth and crust type affect biocrust microbial communities and networks in Mojave Desert, USA. FEMS Microbiol Ecol.

[26] Lan S, Wu L, Zhang D, Hu C (2012). Successional stages of biological soil crusts and their microstructure variability in Shapotou region (China). Environ Earth Sci.

[27] Steven B, Gallegos-Graves LV, Belnap J, Kuske CR (2013). Dryland soil microbial communities display spatial biogeographic patterns associated with soil depth and soil parent material. FEMS Microbiol Ecol.

[28] Kim M, Or D (2017). Hydration status and diurnal trophic interactions shape microbial community function in desert biocrusts. Biogeosciences.

[29] Moreira C Fernandes V, Giraldo‐Silva A, Roush D, Garcia‐Pichel F. Coleofasciculaceae, a monophyletic home for the *Microcoleus steenstrupii* complex and other desiccation‐tolerant filamentous cyanobacteria. J Phycol. 2021;57:1563–79. 10.1111/jpy.13199 34289106

[30] Belnap J, Gardner J (1993). Soil microstructure in soils of the Colorado Plateau: the role of the cyanobacterium *Microcoleus vaginatus*. *West North*. Am Nat.

[31] Ullmann I, Büdel B Ecological Determinants of Species Composition of Biological Soil Crusts on a Landscape Scale. In: Belnap J, Lange OL (eds). *Biological Soil Crusts: Structure, Function, and Management*, 1st ed. 2001. Springer-Verlag, Berlin, pp 203-13.

[32] Lange OL, Belnap J, Reichenberger H, Meyer A (1997). Photosynthesis of green algal soil crust lichens from arid lands in southern Utah, USA: role of water content on light and temperature responses of CO2 exchange. Flora.

[33] Garcia-Pichel F Desert environments: biological soil crusts. *Encycl Environ Microbiol**Vol 6*. 2003. Wiley-Interscience, New York., 1019-23

[34] Pringault O, Garcia-Pichel F (2004). Hydrotaxis of cyanobacteria in desert crusts. Microb Ecol.

[35] Johnson SL, Budinoff CR, Belnap J, Garcia-pichel F (2005). Relevance of ammonium oxidation within biological soil crust communities. Environ Microbiol.

[36] Grondin AE, Johansen JR (1993). Microbial spatial heterogeneity in microbiotic crusts in Colorado National Monument. I. Algae. Gt Basin Nat.

[37] Wheeler CC, Flechtner VR, Johansen JR (1993). Microbial spatial heterogeneity in microbiotic crusts in Colorado National Monument. II. Bacteria. Gt Basin Nat.

[38] Thomas AD, Elliott DR, Hardcastle D, Strong CL, Bullard J, Webster R (2022). Soil biocrusts affect metabolic response to hydration on dunes in west Queensland, Australia. Geoderma.

[39] Nelson C, Giraldo-Silva A, Garcia-Pichel F (2021). A symbiotic nutrient exchange within the cyanosphere microbiome of the biocrust cyanobacterium. Microcoleus vaginatus. ISME J.

[40] Nelson C, Garcia-Pichel F (2021). Beneficial cyanosphere heterotrophs accelerate establishment of cyanobacterial biocrust. Appl Environ Microbiol.

[41] Gomont MM (1892). Monographie des Oscillariées (Nostocacées homocystées). Ann des Sci Nat Bot.

[42] Giraldo-Silva A, Nelson C, Barger N, Garcia-Pichel F (2019). Nursing biocrusts: isolation, cultivation and fitness test of indigenous cyanobacteria. Restor Ecol.

[43] Stanier RY, Kunisawa R, Mandel M, Cohen-Bazire G (1971). Purification and properties of unicellular blue-green algae (order Chroococcales). Bacteriol Rev.

[44] Abràmoff MD, Magalhães PJ, Ram SJ Image Processing with ImageJ. *Optical Imaging Techniques in Cell Biology*. 2006. CRC Press, pp 249-58.

[45] Caporaso JG, Lauber CL, Walters WA, Berg-Lyons D, Lozupone CA, Turnbaugh PJ (2011). Global patterns of 16S rRNA diversity at a depth of millions of sequences per sample. PNAS.

[46] Callahan BJ, McMurdie PJ, Rosen MJ, Han AW, Johnson AJA, Holmes SP (2016). DADA2: High-resolution sample inference from Illumina amplicon data. Nat Methods.

[47] Caporaso JG, Kuczynski J, Stombaugh J, Bittinger K, Bushman FD, Costello EK (2010). QIIME allows analysis of high-throughput community sequencing data. Nat Methods.

[48] Katoh K, Standley DM (2013). MAFFT multiple sequence alignment software version 7: improvements in performance and usability. Mol Biol Evol.

[49] Price MN, Dehal PS, Arkin AP (2010). FastTree 2 – approximately maximum-likelihood trees for large alignments. PLoS One.

[50] Wang Q, Garrity GM, Tiedje JM, Cole JR (2007). Naïve Bayesian classifier for rapid assignment of rRNA sequences into the new bacterial taxonomy. Appl Environ Microbiol.

[51] McDonald D, Price MN, Goodrich J, Nawrocki EP, DeSantis TZ, Probst A (2012). An improved Greengenes taxonomy with explicit ranks for ecological and evolutionary analyses of bacteria and archaea. ISME J.

[52] Garcia‐Pichel F, Zehr JP, Bhattacharya D, Pakrasi HB (2020). What’s in a name? The case of cyanobacteria. J Phycol.

[53] Roush D, Giraldo-Silva A, Garcia-Pichel F (2021). Cydrasil 3, a curated 16S rRNA gene reference package and web app for cyanobacterial phylogenetic placement. Sci Data.

[54] R Development Core Team. R: A language and environment for statistical computing. R Foundation for Statistical Computing, Vienna, Austria. URL http://www.R-project.org/. R Foundation for Statistical Computing, Vienna, Austria. 2020.

[55] Oksanen J, Blanchet FG, Friendly M, Kindt R, Legendre P, McGlinn D (2018). vegan: Community Ecology Package. R package version.

[56] Altschul SF, Gish W, Miller W, Myers EW, Lipman DJ (1990). Basic local alignment search tool. J Mol Biol.

[57] Muyzer G, de Waal EC, Uitterlinden AG (1993). Profiling of complex microbial populations by denaturing gradient gel electrophoresis analysis of polymerase chain reaction-amplified genes coding for 16S rRNA. Appl Environ Microbiol.

[58] Poly F, Monrozier LJ, Bally R (2001). Improvement in the RFLP procedure for studying the diversity of nifH genes in communities of nitrogen fixers in soil. Res Microbiol.

[59] Couradeau E, Karaoz U, Lim HC, Nunes Da Rocha U, Northen T, Brodie E (2016). Bacteria increase arid-land soil surface temperature through the production of sunscreens. Nat Commun.

[60] Maestre FT, Castillo-Monroy AP, Bowker MA, Ochoa-Hueso R (2012). Species richness effects on ecosystem multifunctionality depend on evenness, composition and spatial pattern. J Ecol.

[61] Sorochkina K, Velasco Ayuso S, Garcia-Pichel F (2018). Establishing rates of lateral expansion of cyanobacterial biological soil crusts for optimal restoration. Plant Soil.

[62] Muñoz-Martín MÁ, Becerra-Absalón I, Perona E, Fernández-Valbuena L, Garcia-Pichel F, Mateo P (2019). Cyanobacterial biocrust diversity in Mediterranean ecosystems along a latitudinal and climatic gradient. New Phytol.

[63] Garcia-Pichel F, Loza V, Marusenko Y, Mateo P, Potrafka RM (2013). Temperature drives the continental-scale distribution of key microbes in topsoil communities. Science.

[64] Bates ST, Nash TH, Garcia-Pichel F (2012). Patterns of diversity for fungal assemblages of biological soil crusts from the southwestern United States. Mycologia.

[65] Marusenko Y, Bates ST, Anderson I, Johnson SL, Soule T, Garcia-Pichel F (2013). Ammonia-oxidizing archaea and bacteria are structured by geography in biological soil crusts across North American arid lands. Ecol Process.

[66] Giraldo-Silva A, Fernandes VMC, Bethany J, Garcia-Pichel F (2020). Niche Partitioning with Temperature among Heterocystous Cyanobacteria (*Scytonema* spp., *Nostoc* spp., and *Tolypothrix* spp.) from Biological Soil Crusts. Microorganisms.

[67] Nagy ML, Pérez A, Garcia-Pichel F (2005). The prokaryotic diversity of biological soil crusts in the Sonoran Desert (Organ Pipe Cactus National Monument, AZ). FEMS Microbiol Ecol.

[68] Havrilla CA, Villarreal ML, DiBiase JL, Duniway MC, Barger NN (2020). Ultra‐high‐resolution mapping of biocrusts with Unmanned Aerial Systems. Remote Sens Ecol Conserv.

[69] Campbell EL, Christman H, Meeks JC (2008). DNA Microarray Comparisons of Plant Factor- and Nitrogen Deprivation-Induced Hormogonia Reveal Decision-Making Transcriptional Regulation Patterns in *Nostoc punctiforme*. J Bacteriol.

[70] Garcia-Pichel F, Pringault O (2001). Cyanobacteria track water in desert soils. Nature.

[71] Shrivastava A, Patel VK, Tang Y, Yost SC, Dewhirst FE, Berg HC (2018). Cargo transport shapes the spatial organization of a microbial community. Proc Natl Acad Sci.

[72] Schulz-Bohm K, Gerards S, Hundscheid M, Melenhorst J, de Boer W, Garbeva P (2018). Calling from distance: attraction of soil bacteria by plant root volatiles. ISME J.

[73] Grünbaum D (2012). The logic of ecological patchiness. Interface Focus.

[74] Dubois DM (1975). A model of patchiness for prey—predator plankton populations. Ecol Modell.

[75] Bethany J, Johnson SL, Garcia-Pichel F (2022). High impact of bacterial predation on cyanobacteria in soil biocrusts. Nat Commun.

[76] Fernandes VMC, Machado de Lima NM, Roush D, Rudgers J, Collins SL, Garcia-Pichel F (2018). Exposure to predicted precipitation patterns decreases population size and alters community structure of cyanobacteria in biological soil crusts from the Chihuahuan Desert. Environ Microbiol.

[77] McCann KS (2000). The diversity–stability debate. Nature.

