# Supplementary material for: Spatial self-segregation of pioneer cyanobacterial species drives microbiome organization in biocrusts
Source: ISME Commun. 2022 Nov 16;2:114. doi: 10.1038/s43705-022-00199-0 (PMC9723579; doi:10.1038/s43705-022-00199-0)
Supplement: Supplementary file 1 — Supplementary Information [file 43705_2022_199_MOESM1_ESM.docx]

**Supplementary Information**

**Spatial self-segregation of pioneer cyanobacterial species drives microbiome organization in biocrusts**

Corey Nelson, Ana Giraldo-Silva, Finlay Warsop-Thomas, Ferran Garcia-Pichel

**Supplementary Tables**

**Table S1. Number of bundles assignable to either species by morphology according to cm-sale area samples** Area denomination correspond to those in Figure 1A.

**(SEE TABLE S2 EXCEL FILE)**

**Table S2. Taxonomic and functional characterization of bulk soil and cyanosphere communities.** Taxonomy was assigned to the top 85% most abundant reads in cyanosphere and non-cyanobacterial community of bulk soil using NCBI blastn, to genus-level when possible.

**Table S3.** permANOVA analysis of all cyanospheres on Bray Curtis matrix (10,000 repetitions) showing significance among cyanosphere community compositions by factor. The permANOVA analysis (performed on PRIMER v.7) included the fixed effects of cyanobacterial host, local community of heterotrophs and host x local community.

**Table S4. β-diversity of among single bundles cyanosphere communities of *M. vaginatus* and *Parifilum* sp.**  β-diversity index was calculated for bundles of each species considering the whole dataset. Additionally, bundle communities were randomly subsampled three times (A1, A2, A3) matching the same number of bundles to rule spurious effects of a larger sampling effort in the randomly-sampled bundles. β-diversity values associated to *M. vaginatus* bundles were always smaller than those from *Parafilum* sp. bundles., to genus-level when possible.

**Supplementary Figures**

**Figure S1. Unedited versions of images featured in Figure 1.** No image optimization has been performed and outlines have been removed.
